# Spatio-spectral decomposition of complex eigenmodes in subwavelength nanostructures through transmission matrix analysis

**DOI:** 10.1515/nanoph-2021-0653

**Published:** 2022-01-04

**Authors:** Young-Ho Jin, Juntaek Oh, Wonshik Choi, Myung-Ki Kim

**Affiliations:** KU-KIST Graduate School of Converging Science and Technology, Korea University, Seoul 02841, Republic of Korea; Center for Molecular Spectroscopy and Dynamics, Institute for Basic Science, Seoul 02841, Republic of Korea; Department of Physics, Korea University, Seoul 02841, Republic of Korea

**Keywords:** mode analysis, nano optics, plasmonics, SVD, transmission matrix

## Abstract

Exploiting multiple near-field optical eigenmodes is an effective means of designing, engineering, and extending the functionalities of optical devices. However, the near-field optical eigenmodes of subwavelength plasmonic nanostructures are often highly multiplexed in both spectral and spatial distributions, making it extremely difficult to extract individual eigenmodes. We propose a novel mode analysis method that can resolve individual eigenmodes of subwavelength nanostructures, which are superimposed in conventional methods. A transmission matrix is constructed for each excitation wavelength by obtaining the near-field distributions for various incident angles, and through singular value decomposition, near-field profiles and energy spectra of individual eigenmodes are effectively resolved. By applying transmission matrix analysis to conventional electromagnetic simulations, we clearly resolved a set of orthogonal eigenmodes of single- and double-slot nanoantennas with a slot width of 20 nm. In addition, transmission matrix analysis leads to solutions that can selectively excite specific eigenmodes of nanostructures, allowing selective use of individual eigenmodes.

## 1 Introduction

Optical nanocavities have provided effective channels for strong interaction between light and matter in small spaces owing to their excellent ability to confine light energy in nanometer-scale space. With this excellent property, nanocavities play an important role in driving the development of nano-optical devices, such as nanolasers [1], [2], [3], [4], [5], nanodetectors [6], [7], [8], [9], nanosensors [10], [11], [12], [13], metamaterials [14], [15], [16], [17], [18], and even quantum computing devices [19, 20]. Dielectric-based optical nanocavities, such as photonic crystal cavities, have been actively used with high optical energy density, but their miniaturization is fundamentally limited by the diffraction limit. In contrast, metal-based plasmonic nanocavities allow the realization of subwavelength nanocavities beyond the diffraction limit by utilizing surface plasmon polaritons (SPPs). In particular, plasmonic nanocavities composed of a few small metallic segments (e.g., nanorods) [21], [22], [23] or a few small pores in metallic films (e.g., nanoslots) [24], [25], [26] act as nanoantennas, enabling free-propagating light to be efficiently converted into strongly enhanced subwavelength optical fields, and vice versa. Far-to-near-field coupling via nanoantennas can generate very strong and localized electric fields, which are essential for applications such as single-molecule detection [27], [28], [29], Raman spectroscopy [30], [31], [32], and single-photon generation [33], [34], [35]. Recently, functional engineering of nanoantennas using near-field engineering with structural deformation [36], [37], [38] or near-field coupling with other antennas [39], [40], [41] has been actively studied for practical applications. Further exploitation of high-order near-field eigenmodes present in nanoantennas along with accurate mode analysis will provide a potent opportunity to control antenna functionalities in versatile ways. However, because the high-order eigenmodes of subwavelength nanoantennas are spatially and spectrally superimposed on each other in a complex manner, their accurate analysis and active utilization are significant challenges both simulation-wise and experimentally. In a recent study, we experimentally demonstrated a new near-field imaging method that can resolve the near-field eigenmodes of double-slot nanoantennas through near-field scanning optical microscopy (NSOM) measurements [42]. In that experiment, we constructed a fully phase-referenced far-to-near-field transmission matrix (T-matrix) at a single wavelength and observed the decomposed near-field eigenmodes through singular value decomposition (SVD) of the measured matrix. These experimental observations indicated the potential to develop a novel mode decomposition method that can effectively resolve the spatio-spectrally complex eigenmodes of subwavelength nanostructures not only experimentally but also through simulations.

In this study, we developed a new mode analysis method based on a T-matrix, which can effectively resolve spatio-spectrally multiplexed eigenmodes of subwavelength nanostructures. We consider the construction of a wavelength-dependent T-matrix from near-field distributions for various wavevectors (or angles) of incidence beams at each excitation wavelength. We provide a theoretical framework for the energy spectrum and near-field profile of each eigenmode superimposed by the other eigenmodes to be resolved through SVD of the T-matrix. In addition, by applying the T-matrix-based mode analysis method to a finite-difference time-domain (FDTD) simulation, we achieved a clear decomposition of energy spectra and near-field profiles of high-order eigenmodes of single- and double-slot nanoantennas with a slot width of 20 nm. Moreover, the T-matrix-based mode analysis method leads to solutions of incident beam wavefronts that can selectively excite specific eigenmodes of nanostructures, allowing us to selectively use individual eigenmodes of subwavelength nanostructures.

## 2 T-matrix-based mode analysis

**Figure 1:**
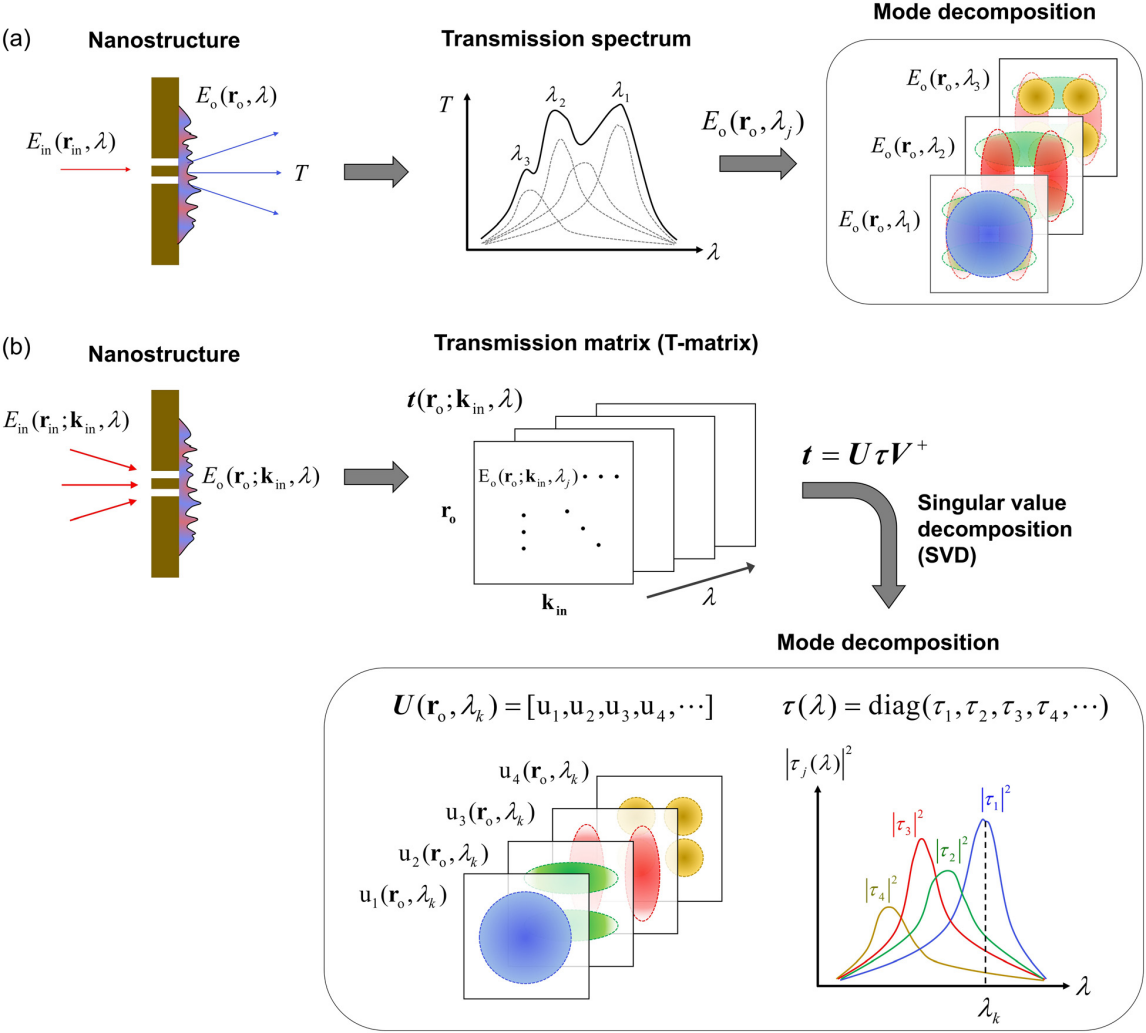
Comparison of mode analysis methods based on transmission spectrum and transmission matrix (T-matrix). (a) Process flow diagram of a conventional single-channel (i.e., single specific **k**_in_) transmission measurement analysis. Transmission spectra (middle panel) and field profiles (right panel) of the nano-optical system obtained through conventional single-channel transmission analysis. (b) Process flow diagram of a multi-channel (i.e., multiple **k**_in_) measurement for the T-matrix-based mode analysis. The transmission matrix ***t*** (**r**_o_; **k**_in_, *λ*) is reconstructed for each wavelength from multi-channel measurements. Decomposition of the matrices $t\left(r_{\text{o}};k_{\text{in}},\lambda \right)=U\tau V^{†}$ through singular value decomposition (SVD). Field profiles and energy spectra of each eigenmode of the nano-optical system extracted from ***U*** and ***τ***, respectively.

Figure 1a and b show schematic flow diagrams comparing the conventional transmission spectrum-based mode analysis and proposed T-matrix-based mode analysis methods, respectively. In the conventional method in Figure 1a, a broadband incident beam $E_{\text{in}}\left(r_{\text{in}},\lambda \right)$ is applied to the nanostructure at a specific angle to obtain a transmission spectrum *T*(*λ*) (solid black curve in the middle panel), where **r**_in_ and *λ* are the spatial coordinate of the input plane of the nanostructure and the input wavelength, respectively, and based on this curve, the resonance eigenfrequencies are estimated (e.g., $\lambda_{j}=\left\{\lambda_{1},\lambda_{2},\lambda_{3}\right\}$ in the middle panel of Figure 1a). Here, the spatial field distribution $E_{\text{o}}\left(r_{\text{o}},\lambda_{j}\right)$ of each eigenmode is resolved through spectral filtering of the measured complex near-field $E_{\text{o}}\left(r_{\text{o}},\lambda \right)$ around the estimated eigenfrequency, where **r**_o_ is the spatial coordinate of the output plane of the nanostructure. In this method, the spectrum of each mode must be distinct from those of the other modes for precise mapping of the spatial field profile of the corresponding mode. That is, as the spectral linewidth (δ*λ*) of each mode becomes sufficiently narrower than the free-spectral range (Δ*λ*) between adjacent modes (or δ*λ* ≪ Δ*λ*), the mode analysis becomes more accurate. Typically, when the size of the optical resonator is increased (or decreased), δ*λ* and Δ*λ* are simultaneously narrowed (or widened) due to the relation of $\delta \lambda =\left(\lambda^{2}/4\pi l\right)\cdot \text{ln}\left[R_{1}R_{2}\left(1-L_{\text{RT}}\right)\right]$ [43] and $\Delta \lambda =\lambda^{2}/n_{\text{g}}l$ [44], where *R*_1_ and *R*_2_ are the intensity reflectances of the two resonator mirrors, *L*_RT_ is the intrinsic round-trip loss of the resonator, *n*_g_ is the group index of the media within the resonator, and *l* is the length of the resonator. So, the ratios of δ*λ* and Δ*λ* to the size (*l*) of the resonator do not differ significantly. However, in subwavelength plasmonic nanostructures, δ*λ* is considerably widened due to large absorption loss by metals and strong radiation loss due to small sizes, whereas Δ*λ* is narrowed due to the high effective refractive index of the plasmonic modes. As a result, a condition of δ*λ* ≫ Δ*λ* is created, which is unfavorable for mode decomposition. Specifically, spatial field profiles of the neighboring modes are overlapped with that of the mode of interest, as illustrated in the right panel of Figure 1a. As such, conventional transmission spectrum-based mode analysis has significant limitations in analyzing the resonant eigenmodes of subwavelength plasmonic nanostructures.

In contrast, the proposed mode analysis method is able to effectively decompose complex resonant eigenmodes of subwavelength nanostructures based on the T-matrix, which contains both spatial information of transmitted waves and wavevector information of incident beams (Figure 1b). The T-matrix is typically represented as a set of complex field maps at the output plane of the device for free-mode excitations at the input plane. It has been used to analyze and utilize the coherent linear interaction between light and arbitrary devices including disordered media [45], [46], [47], [48]. Here, the T-matrix $t\left(r_{\text{o}};k_{\text{in}},\lambda \right)$ for a wavelength *λ* is constructed by applying a planewave $E_{\text{in}}\left(r_{\text{in}};k_{\text{in}},\lambda \right)$ at a specific angle (or a specific in-plane wavevector **k**_in_) to the nanostructure and arranging the transmitted wave $E_{\text{o}}\left(r_{\text{o}};k_{\text{in}},\lambda \right)$ obtained from the surface of the nanostructure. The transmitted wave ***E***_o_ can be formulated as

(1) $$E_{\text{o}}\left(r_{\text{o}};k_{\text{in}},\lambda \right)=\sum c_{j}\left(k_{\text{in}},\lambda \right)\tau_{j}\left(\lambda \right)u_{j}\left(r_{\text{o}},\lambda \right)\text{,}$$

where $c_{j}\left(k_{\text{in}},\lambda \right)$ , *τ*_*j*_(*λ*), and $u_{j}\left(r_{\text{o}},\lambda \right)$ are the coupling coefficient, transmission coefficient, and complex-field distribution of the *j*th eigenmode at the output plane, respectively. $c_{j}\left(k_{\text{in}},\lambda \right)$ is given by

(2) $$c_{j}\left(k_{\text{in}},\lambda \right)=〈v_{j}\left(r_{\text{in}},\lambda \right)E_{\text{in}}^{*}\left(r_{\text{in}};k_{\text{in}},\lambda \right)〉r_{\text{in}}\text{,}$$

where $v_{j}\left(r_{\text{in}},\lambda \right)$ is the complex-field distribution of the input source for excitation of the *j*th eigenmode. <> indicates the average of the elements within the parentheses with respect to the subscript. The T-matrix $t\left(r_{\text{o}};k_{\text{in}},\lambda \right)$ at a specific wavelength *λ* is constructed by assigning $E_{\text{o}}\left(r_{\text{o}};k_{\text{in}},\lambda \right)$ to the column and row indices of ***t*** corresponding to **k**_in_ and **r**_o_, respectively. To decompose the eigenmodes from ***t***, we apply SVD, i.e., $t\left(r_{\text{o}};k_{\text{in}},\lambda \right)=U\tau V^{†}$ , where † indicates the conjugate transpose of a matrix, and ***τ*** is a diagonal matrix whose diagonal elements are non-negative real numbers referred to as singular values. The squares of these singular values are the eigenvalues of the matrix ***t*** ^†^ ***t***. In this manner, we can extract ${\text{v}}_{j}\left(r_{\text{in}},\lambda \right)$ from the *j*th the column of ***V***, **u**_*j*_(**r**_o_, *λ*) from the *j*th column of ***U***, and ***τ***_*j*_(*λ*) from the diagonal element of ***τ***. In other words, spatial field profiles of individual eigenmodes (**u**_*j*_(**r**_o_, *λ*)) can be separately obtained, as illustrated in Figure 1b. Plotting the eigenvalue ${\left|\tau_{j}\left(\lambda \right)\right|}^{2}$ as a function of wavelength *λ* gives the energy spectrum of the *j*th eigenmode (Figure 1b). Therefore, the T-matrix-based mode analysis method gives the energy spectrum of each mode, whereas the transmission spectrum-based analysis method shows the complex spectrum in which multiple modes are superimposed. Moreover, the T-matrix-based mode analysis method also provides the complex-field distribution ${\text{v}}_{j}\left(r_{\text{in}},\lambda \right)$ of the input source, which can excite a specific eigenmode of the nanostructure. Essentially, the proposed T-matrix-based mode analysis demultiplexes eigenmodes that are spatio-spectrally multiplexed in conventional mode analysis. Therefore, the proposed method is a notable breakthrough that can overcome the limitations of conventional mode analysis of plasmonic nanostructures at subwavelength scale.

## 3 Mode analysis of single-slot nanoantenna

**Figure 2:**
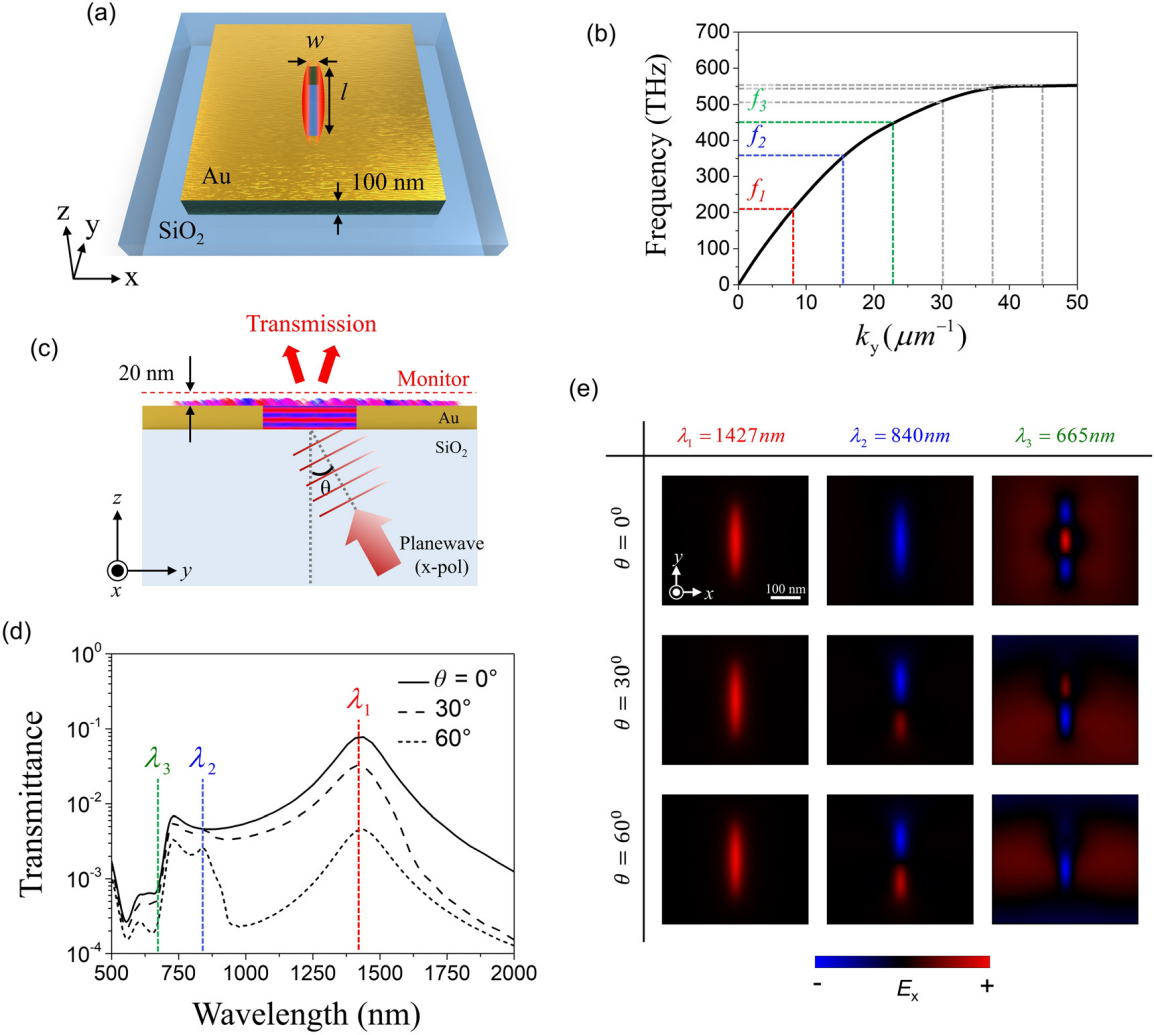
Transmission spectrum-based mode analysis for single-slot nanoantenna. (a) Schematic of single-slot nanoantenna with a width (*w*) of 20 nm and length (*l*) of 300 nm. (b) Dispersion relation of gold–air–gold waveguide mode propagating along *y*-direction. Red, blue, and green dashed lines indicate 1st, 2nd, and 3rd resonant modes, respectively. (c) Schematic of single-channel measurement simulation condition of a single-slot nanoantenna by changing the incident angle (*θ*). (d) Transmittance spectrum of a single-slot nanoantenna by adjusting incident angle. Solid, dashed, and dotted curves indicate transmitted spectra for the incident angles of 0, 30, and 60°, respectively. Red, blue, and green dashed lines indicate the 1st, 2nd, and 3rd modes, respectively. (e) Real *E*_*x*_ field profiles of single-slot nanoantenna for 0, 30, and 60 degrees of incident angle at 1st (1427 nm), 2nd (840 nm), and 3rd (665 nm) modes.

To validate the proposed T-matrix-based mode analysis method, we analyzed a simple single-slot nanoantenna through FDTD simulation (Supplementary Material). Figure 2a shows the plasmonic nanoantenna in which a single air-slot with a width (*w*) of 20 nm and length (*l*) of 300 nm was introduced onto a 100 nm-thick gold film on a glass substrate. This structure supports a metal–insulator–metal (MIM) SPP mode that propagates in the *y* direction with polarization in the *x* direction, and the dispersion relation of this propagation mode was calculated as shown in Figure 2b. In this dispersion, the slope of the dispersion curve decreased with increasing frequency, causing both the phase and group velocity of the SPPs to slow down. The cutoff frequency was calculated to be 550 THz (or *λ*_cutoff_ =** **545 nm). In the antenna structure of Figure 2a, the length of the air-slot in the *y*-direction is finite at 300 nm, so the *y*-component of the wavevector ***k***_*y*_ has a discrete value of m×(π/l) , where *m* is an integer. Thus, the 1st, 2nd, and 3rd eigenmodes of this antenna structure have resonant eigenfrequencies ***f***_*m*_ of 210, 357, and 451 THz (or resonant wavelengths of 1427, 840, and 665 nm) corresponding to ***k***_*y*_ with *m* of 1, 2, and 3, respectively. In particular, as the order of the mode increases, the free-spectral range between eigenfrequencies ( $\Delta f=c\Delta \lambda /\lambda^{2}$ ) further narrows.

We first attempted to decompose the eigenmodes of a single-slot nanoantenna using the conventional transmission spectrum. To measure the transmission spectrum, we sent a planar wave at an incident angle of *θ* with polarization in the *x*-direction from a glass substrate to the nanoantenna and obtained the complex near-field distribution in the plane 20 nm above the gold surface, as shown in Figure 2c. Figure 2d shows the measured transmittance spectra at *θ* = 0°, 30°, and 60°. Through these spectrum curves, the resonant wavelength of the 1st mode was estimated, but as the order of the mode increased, it became difficult to distinguish individual eigenmodes due to the condition of δ*λ* ≫ Δ*λ*. In particular, in the case of a normally incident beam (*θ* = 0°), only the 1st and 3rd modes were excited, and as the angle increased (*θ* = 30° and 60°), the second mode appeared weakly mixed with the spectra of the 1st and 3rd modes. Figure 2e shows the spatial field distributions of the 1st, 2nd, and 3rd eigenmodes for the incident beams at three different angles (*θ* = 0°, 30°, and 60°), which were obtained by spectrally filtering the complex near-field distribution in the vicinity of the resonant wavelength of each eigenmode (*λ* = 1427, 840, and 665 nm, respectively; Supplementary Material). For a 0° incident beam, the distinct spatial near-field distributions of the 1st and 3rd eigenmodes were visible, whereas the 2nd mode was not observed. In the cases of the 30° and 60° incident beams, the 1st and 2nd modes were resolved, but the 3rd mode was either mixed with the 2nd mode or invisible. These results show that it is difficult to accurately distinguish the spatial field profiles and resonant wavelengths of near-field eigenmodes in subwavelength plasmonic nanostructures through conventional analysis method based on the transmission spectrum.

**Figure 3:**
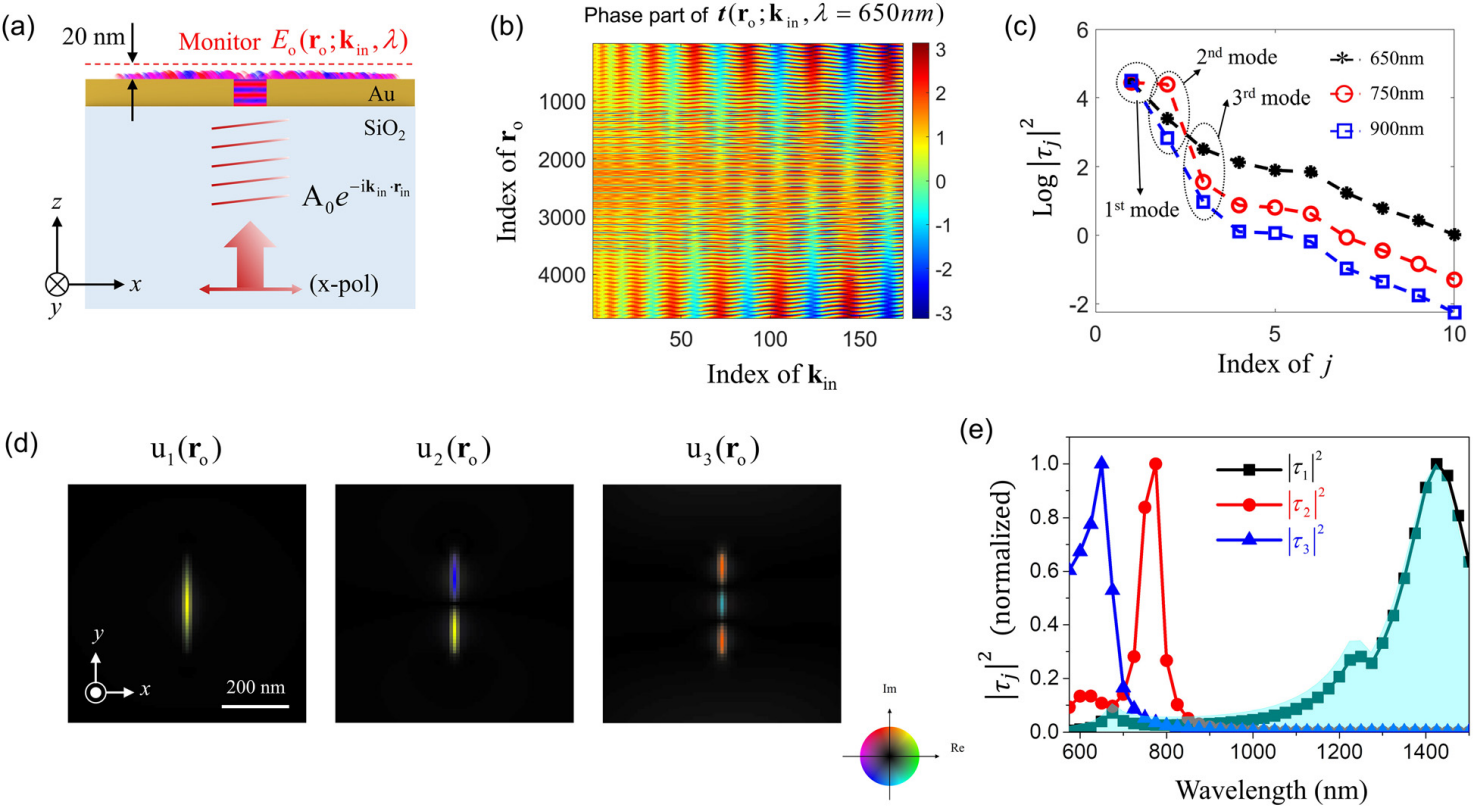
T-matrix-based mode analysis for single-slot nanoantenna. (a) Simulation schematic diagram of T-matrix-based mode analysis. (b) Phase part of T-matrix constructed from the near-field complex-field maps obtained from the monitor in (a) at wavelength 650 nm. The column index indicates **k**_in_ sorted in increasing order of magnitude. The row index describes **r**_o_ sorted in increasing order of its magnitude; only the phase part of the T-matrix is shown. (c) Squares of singular values, ${\left|\tau_{j}\left(\lambda \right)\right|}^{2}$ , of T-matrices sorted in descending order. The first three eigenvalues are related to the meaningful eigenmodes of the single-slot nanoantenna, whose indices are indicated by black dashed circles. Black, red, and blue graphs indicate the energy distributions from the 650, 750, and 900 nm wavelengths, respectively. (d) Complex-field maps of eigenmodes obtained from the first three columns of the unitary matrix **U** of the T-matrix in (b). Circular color map: real and imaginary values of the complex field. (e) Normalized energy spectrum of each individual eigenmode. The light-blue window indicates normalized transmittance with normally incident beam from Figure 2d.

On the contrary, the T-matrix-based mode analysis method allows us to effectively resolve spatio-spectrally multiplexed eigenmodes in subwavelength nanostructures. We obtained the T-matrix ***t*** (**r**_o_; **k**_in_, *λ*) by applying input sources with various *k*-vectors $k_{\text{in}}=k\text{ sin }\theta_{\text{in}}$ (174 total from $\left|\theta_{\text{in}}\right|=0{}^{\circ}$ to sin^−1^0.6; Supplementary Material) and acquiring the corresponding complex near-field distributions $E_{\text{o}}\left(r_{\text{o}};k_{\text{in}},\lambda \right)$ in the plane 20 nm above the gold surface (Figure 3a). Here, the T-matrix was constructed for each wavelength *λ* by assigning $E_{\text{o}}\left(r_{\text{o}};k_{\text{in}},\lambda \right)$ to the column and row indices associated with **k**_in_ and **r**_o_, respectively. Figure 3b shows the measured phase part of the T-matrix for a wavelength of 650 nm as a representative example (Supplementary Material). As shown in this figure, the spatial phase distribution ***E***_o_(**r**_o_) varied with the incident beam wavevector **k**_in_, which is due to the angle-dependent mode coupling $c_{j}\left(k_{\text{in}},\lambda \right)$ . The eigenmodes of the measured T-matrix $t\left(r_{\text{o}};k_{\text{in}},\lambda \right)$ could be decomposed by applying SVD, as shown in Figure 1b, and we extracted eigenvalues (or squares of singular values ${\left|\tau_{j}\left(\lambda \right)\right|}^{2}$ ) for each wavelength, which were sorted in descending order, as shown in Figure 3c. Here, the eigenvalue of the lowest-order mode was the highest for all wavelengths due to its high coupling efficiency. The complex-field distributions ${\text{u}}_{j}\left(r_{\text{o}}\right)$ of the three lowest-order eigenmodes decomposed at a wavelength of 650 nm are shown in Figure 3d. Compared with Figure 2e, Figure 3d shows clear distinction between individual mode. Furthermore, plotting the eigenvalue ${\left|\tau_{j}\left(\lambda \right)\right|}^{2}$ as a function of wavelength *λ* gives the energy spectrum of the *j*th eigenmode (Figure 3e), which resolves the spectra of the three eigenmodes very accurately compared to Figure 2d. As a point of reference, the spectrum of Figure 2d at *θ* = 0° is shown by the area filled with light blue, which almost coincides with the decomposed ${\left|\tau_{j=1}\left(\lambda \right)\right|}^{2}$ spectrum with almost no signatures of the 2nd- and 3rd-order modes. On the contrary, the hidden 2nd- and 3rd-order modes were clearly resolved by the respective plots of ${\left|\tau_{j=2}\left(\lambda \right)\right|}^{2}$ and ${\left|\tau_{j=3}\left(\lambda \right)\right|}^{2}$ through T-matrix-based mode analysis. The spectral centers of the identified modes and tendency of the spectral width to narrow, or equivalently of the quality factor (Q-factor) to increase, with an increase of mode order agree well with the theoretical prediction. These results support that the T-matrix-based mode analysis has an excellent ability to resolve highly multiplexed complex eigenmodes of subwavelength nanostructures. In this simulation, however, we could not fully resolve the fourth or higher-order eigenmodes through SVD of the T-matrix because the noise generated in the simulation was larger than the energy of the fourth or higher-order eigenmodes of the nanoantenna. In particular, weak reflections of perfectly matched layers (open boundary layers) placed around the simulation domain accumulated energy inside the simulation, which became larger than the energy of the fourth or higher-order eigenmodes of the nanoantenna. Essentially, systematic and measurement noise will limit the number of higher-order eigenmodes that can be resolved through the T-matrix-based mode analysis method, not only in simulations but also in experiments [42].

## 4 Mode analysis of double-slot nanoantenna

**Figure 4:**
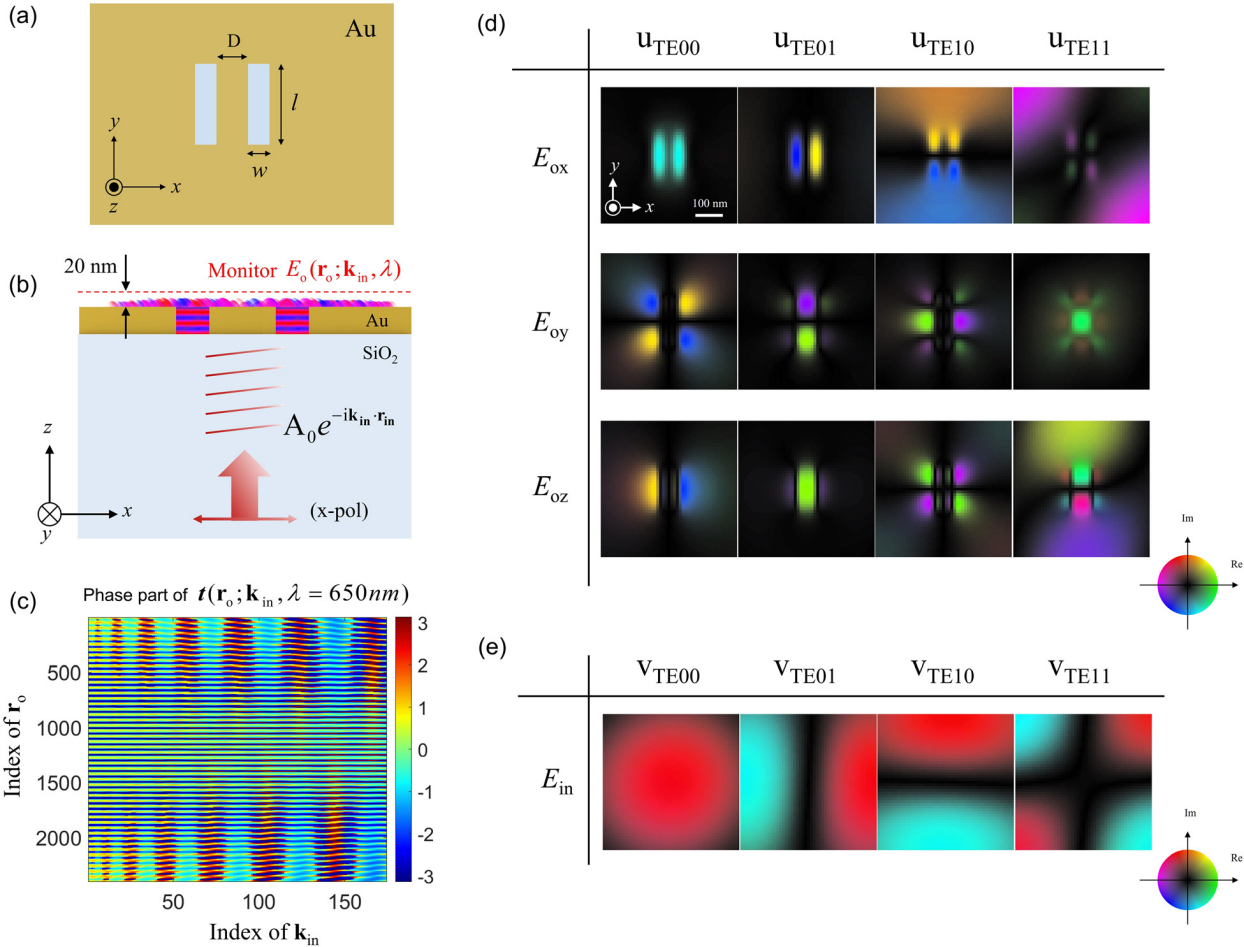
T-matrix-based mode analysis for double-slot nanoantenna. (a) and (b) Simulation schematics of double-slot nanoantenna in *xy*- and *zx*-planes, respectively. Here, the slot width (*w*), length (*l*), and spacing (*D*) are 20, 160, and 50 nm, respectively. (c) Phase part of T-matrix constructed from the near-field complex-field maps obtained at wavelength 650 nm. The column index indicates **k**_in_ sorted in increasing order of its magnitude. The row index describes **r**_o_ sorted in increasing order of its magnitude. Only the phase part of the T-matrix is shown. (d) Electric fields of *x*, *y*, and *z* components of the double-slot nanoantenna. (e) Electric field of input beam for the excitation of each eigenmode. Circular color maps: real and imaginary values of the complex field.

To apply the T-matrix-based mode analysis to more complex structures, we introduced the double-slot nanoantenna shown in Figure 4a, with slot width (*w*), length (*l*), and spacing (*D*) of 20 nm, 160 nm, and 50 nm, respectively, all of which are much smaller than the wavelength. Similar to Figure 3a, we obtained the T-matrix by applying input sources with various *k*-vectors (174 total from $\left|\theta_{\text{in}}\right|=0{}^{\circ}$ to sin^−1^0.6) and acquiring the corresponding complex near-field distributions in the plane 20 nm above the gold surface (Figure 4b). Figure 4c shows the phase part of the measured T-matrix for a wavelength of 650 nm. After applying SVD to the measured T-matrix (Supplementary Material), the complex-field distributions **u**_*j*_(**r**_o_) for the *x*, *y*, and *z* components of the four lowest-order eigenmodes (TE00, TE01, TE10, and TE11 modes) were decomposed (Figure 4d). These four decomposed eigenmodes were distinguished through the symmetry conditions for the *x*-component of the electric field (*E*_o*x*_). In the TE00 mode, *E*_o*x*_ has even symmetry with respect to both the *yz*-plane (at *x* = 0) and *xz*-plane (at *y* = 0), and this symmetric condition is consistent with that of the incident planewave with *x*-polarization. Meanwhile, *E*_o*x*_ of the TE01 mode has odd and even symmetry, and the TE10 mode has even and odd symmetry with respect to the *yz*- and *xz*-planes, respectively. For the TE11 mode, *E*_o*x*_ has odd symmetry for both the *yz*- and *xz*-planes. Furthermore, we were able to inversely calculate the distribution of the incident beam ***E***_in_, which can selectively excite only a specific eigenmode from the ***V*** ^†^ matrix decomposed through SVD. These results allow us to find the condition of the incident beam that can individually excite only the desired eigenmode.

**Figure 5:**
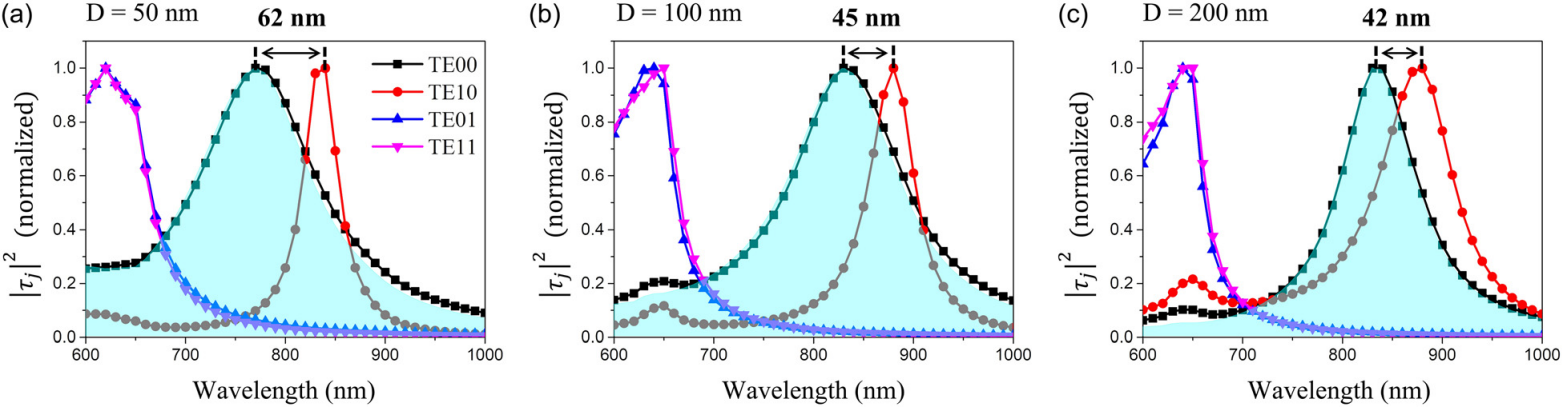
Energy spectra of double-slot nanoantenna depending on slot spacing. (a–c) Individual energy spectra for the slot spacing of double-slot nanoantennas: *D* = 50, 100, and 200 nm, respectively. The light-blue widow indicates normalized transmittance of double-slot nanoantennas with normally incident beam.

Finally, we attempted to decompose the spectral splitting between the fundamental even (TE00) and odd (TE10) symmetric modes according to a small change of *D* (50, 100, and 200 nm) using T-matrix-based mode analysis. Figure 5a–c show the energy spectra for the double-slot nanoantennas with *D* = 50, 100, and 200 nm, respectively, which were obtained by plotting the eigenvalues as a function of wavelength *λ* for each eigenmode. Here, the areas filled with light blue represent the transmission spectra for the incidence beam at *θ* = 0°. From these results, we can see that the transmission spectrum (area filled with light blue) and the energy spectrum of the TE00 mode (black curve) are very similar. This is because the incident beam at *θ* = 0° has high coupling efficiency with the TE00 mode due to their similar field symmetric conditions, as shown in Figure 4d. This means that spectral decomposition between the TE00 and TE10 modes is very difficult with only the transmission spectrum. However, the energy spectra decomposed based on the T-matrix analysis could resolve small spectral splitting between the central wavelengths of the TE00 and TE10 modes (62, 45, and 42 nm) for the structures with *D* = 50, 100, and 200 nm, respectively. Furthermore, subtle changes in the spectral broadness (or Q-factors) of the TE00 and TE10 modes depending on *D* were also clearly observed, which were attributed to the difference in coupling strength between the nano-slots [49, 50]. This suggests that the T-matrix-based mode analysis method provides advanced spectral resolution as well as spatial resolution for the mode decomposition of complex subwavelength nanostructures, exceeding the limitations of conventional mode analysis.

## 5 Conclusions

In conclusion, we proposed a T-matrix-based mode analysis method that can effectively extract high-order near-field eigenmodes of subwavelength plasmonic nanostructures, which are inaccessible with conventional mode analysis methods. In particular, from the T-matrices constructed for various wavelengths, the spectra of individual eigenmodes, which are superimposed in conventional mode analysis methods, were clearly resolved along with their near-field profiles. Essentially, the T-matrix enables individual eigenmodes to be discriminated by exploiting the fact that high-order eigenmodes are driven differently depending on various incident phase conditions. This provides a new opportunity to resolve fine modal details of nanostructures with sizes much smaller than the wavelength. Furthermore, our approach can be experimentally combined with NSOM modalities [42], help extract various pieces of near-field eigenmode information, such as electrical and magnetic near-field vector components, and time/frequency resolved measurements [51], [52], [53], [54], [55]. This could provide new insights for designing and engineering the functionality of subwavelength plasmonic devices with active combination of high-order near-field optical eigenmodes.

## Supplementary Material

Supplementary Material
