# Supplementary material for: Spatio-spectral decomposition of complex eigenmodes in subwavelength nanostructures through transmission matrix analysis
Source: Nanophotonics. 2022 Jan 4;11(9):2149–58. doi: 10.1515/nanoph-2021-0653 (PMC11501628; doi:10.1515/nanoph-2021-0653)
Supplement: Supplementary file 1 — Supplementary Material [file j_nanoph-2021-0653_suppl.pdf]

Supplementary Information

Spatio-spectral decomposition of complex eigenmodes in subwavelength nanostructures through transmission matrix analysis

Young-Ho Jin[1], Juntaek Oh[2,3], Wonshik Choi[2,3,*], and Myung-Ki Kim[1,*]

[1]*KU-KIST Graduate School of Converging Science and Technology, Korea University, Seoul 02841, Republic of Korea*

[2]*Center for Molecular Spectroscopy and Dynamics, Institute for Basic Science, Seoul 02841, Republic of Korea*

[3]*Department of Physics, Korea University, Seoul 02841, Republic of Korea*

[*]*e-mail: wonshik@korea.ac.kr and rokmk@korea.ac.kr*

Supplementary Note 1: Simulation details

We employed the FDTD method (Lumerical Solutions, Inc.; https://www.lumerical.com/) to simulate the optical response of the nanoantennas. The single and double air slots were introduced onto a 100-nm-thick gold film on an SiO_2 substrate. The dielectric constants of gold were taken from Johnson & Cristy's experimental study [S1], and the refractive indices of glass and background were set as 1.5 and 1.0, respectively. To ensure the precision of the simulation, we defined meshes around the nanoantennas as Δx=10 nm, Δy=10 nm, and Δz=10 nm. We used an x-polarization planar wave source (174 input angles) and detected the near-field (E_x, E_y, E_z) in the plane 20 nm above the surface of nanoantennas. We obtained complex near-field distributions by scanning input angles and wavelengths to construct wavelength-dependent T-matrices.

[S1] P. B. Johnson, and R. W. Christy, "Optical constants of the noble metals," Phys. Rev. B **6**(12), 4370–4379 (1972).

Supplementary Note 2: Near-fields of single-slot nanoantenna resolved by transmission spectrum analysis

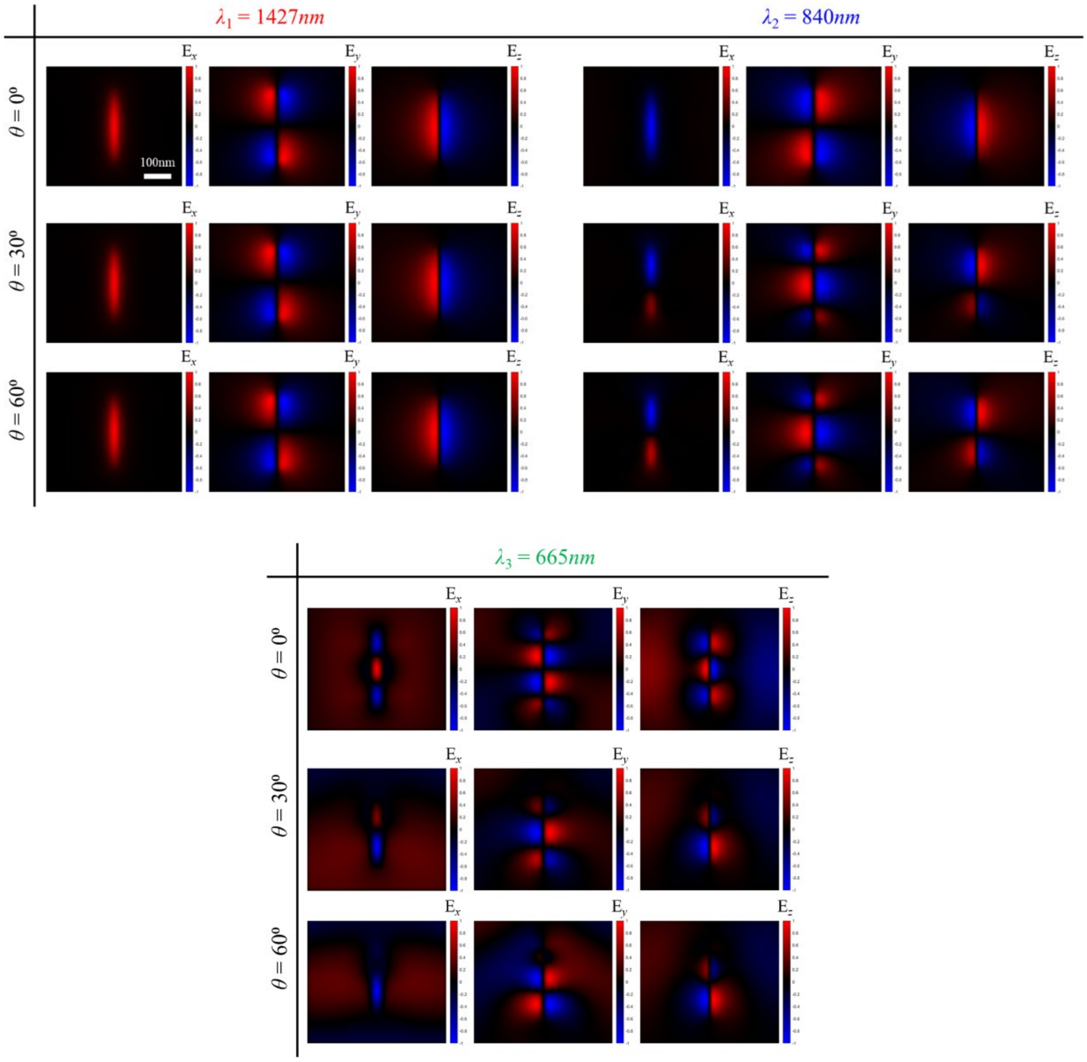

Figure S1. Near-fields of single-slot nanoantenna resolved by transmission spectrum-based mode analysis: Normalized real E_x, E_y, and E_z field profiles of single-slot nanoantenna for 0, 30, and 60 degrees of incident angle at 1st (1427 nm), 2nd (840 nm), and 3rd (665 nm) modes.

Supplementary Note 3: Angles of incident beam to construct T-matrix

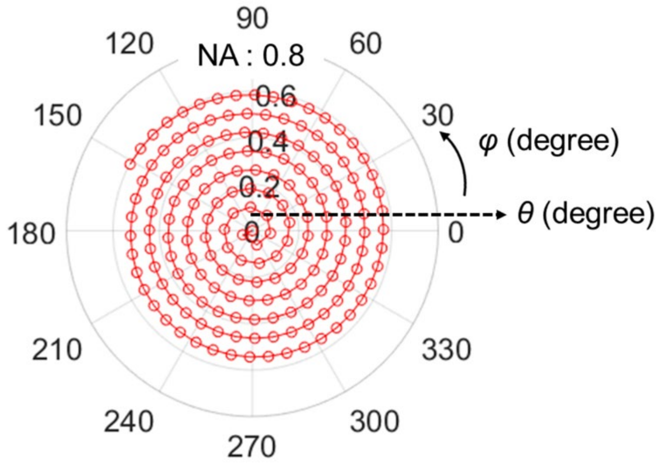

Figure S2. Angles of incident beam to construct T-matrix: We obtained the T-matrix by applying input sources with various k-vectors $\mathbf{k_{in}} = \mathbf{k}\,\sin\theta_{\mathrm{in}}$ (174 total from $|\theta_{\mathrm{in}}| = 0°$ to $\sin^{-1}0.6$) and acquiring the corresponding complex near-field distributions. Circular dots represent 174 $\mathbf{k_{in}}/\mathrm{k}\;(=\left(\mathrm{k}_x^{\mathrm{in}}, \mathrm{k}_y^{\mathrm{in}}\right)/\mathrm{k})$ used in the simulation within a numerical aperture of 0.6.

Supplementary Note 4: Constructed T-matrix of single-slot nanoantenna

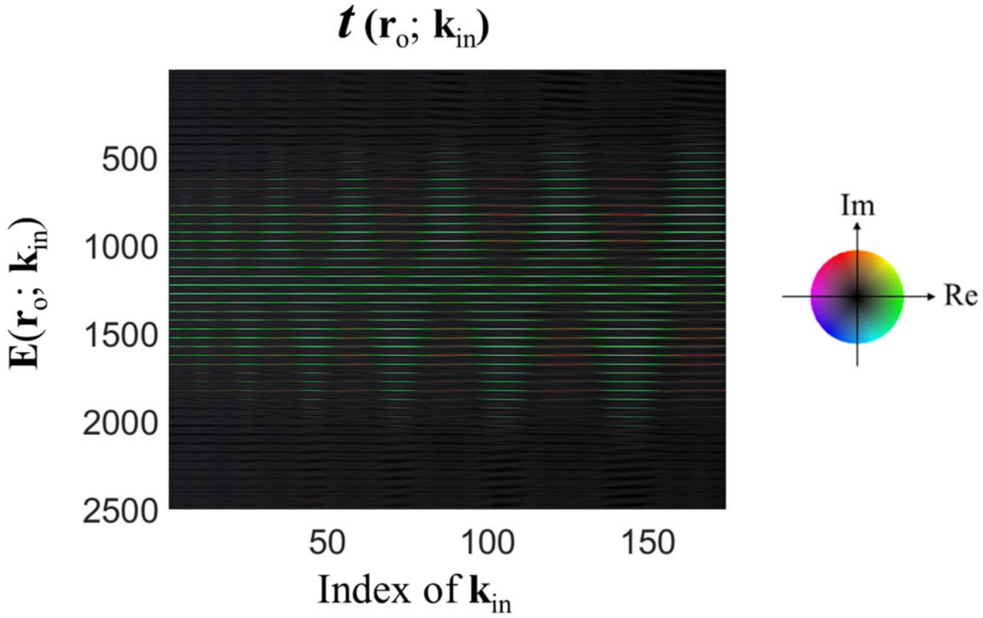

Figure S3. Constructed T-matrix of single-slot nanoantenna: T-matrix constructed from the near-field complex-field maps obtained from the monitor in Fig. 3(a) at wavelength 650 nm by assigning E_{o} to the column and row indices associated with $\mathbf{k}_{\mathrm{in}}$ and $\mathbf{r}_{\mathrm{o}}$, respectively. Circular color map: real and imaginary values of the complex field.

Supplementary Note 5: Constructed T-matrix of double-slot nanoantenna

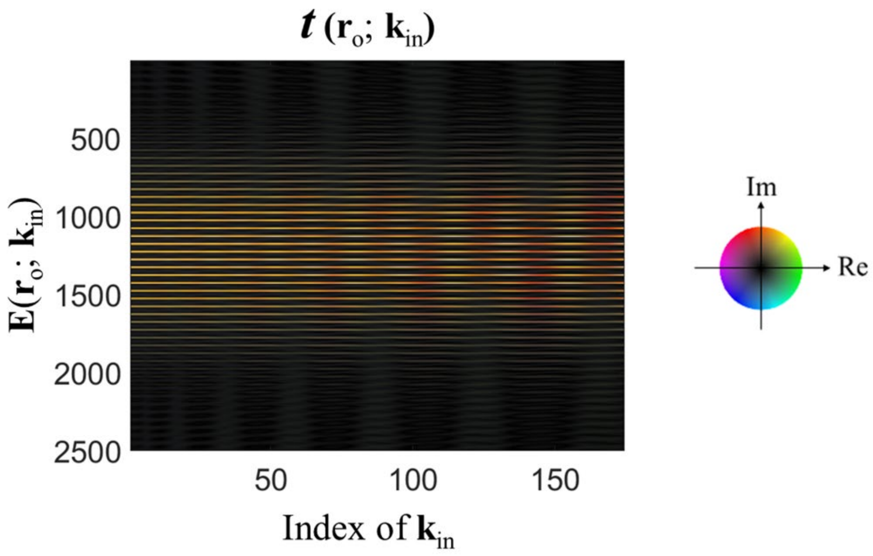

Figure S4. Constructed T-matrix of double-slot nanoantenna: T-matrix constructed from the near-field complex-field maps obtained from the monitor in Fig. 4(b) at wavelength 650 nm by assigning E_{o} to the column and row indices associated with $\mathbf{k}_{\mathrm{in}}$ and $\mathbf{r}_{\mathrm{o}}$, respectively. Circular color map: real and imaginary values of the complex field.

Supplementary Note 6: Singular value decomposition of T-matrix of double-slot nanoantenna

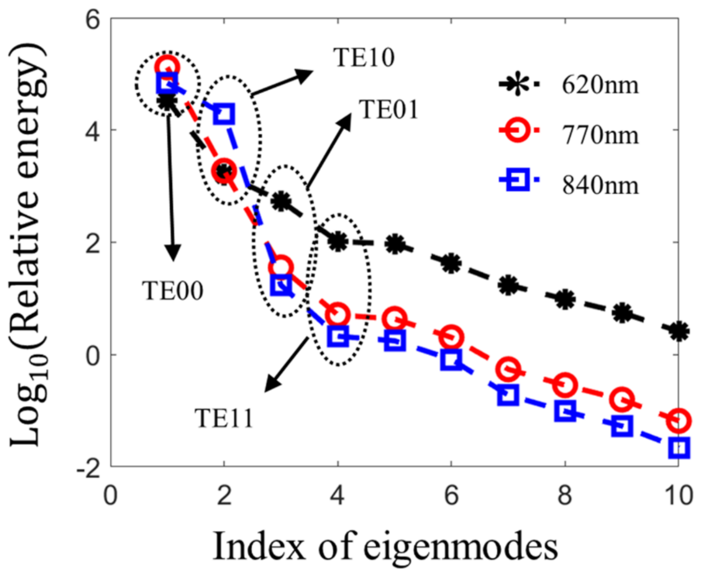

Figure S5. Singular value decomposition of T-matrix of double-slot nanoantenna: Squares of singular values of T-matrices sorted in descending order. The first four eigenvalues are related to the meaningful eigenmodes (TE00, TE10, TE01, and TE11 modes) of the double-slot nanoantenna, whose indices are indicated by black dashed circles. Black, red, and blue graphs indicate the energy distributions from the 620, 770, and 840 nm wavelengths, respectively.

Supplementary Note 7: Energy spectra of double-slot nanoantenna depending on slot spacing

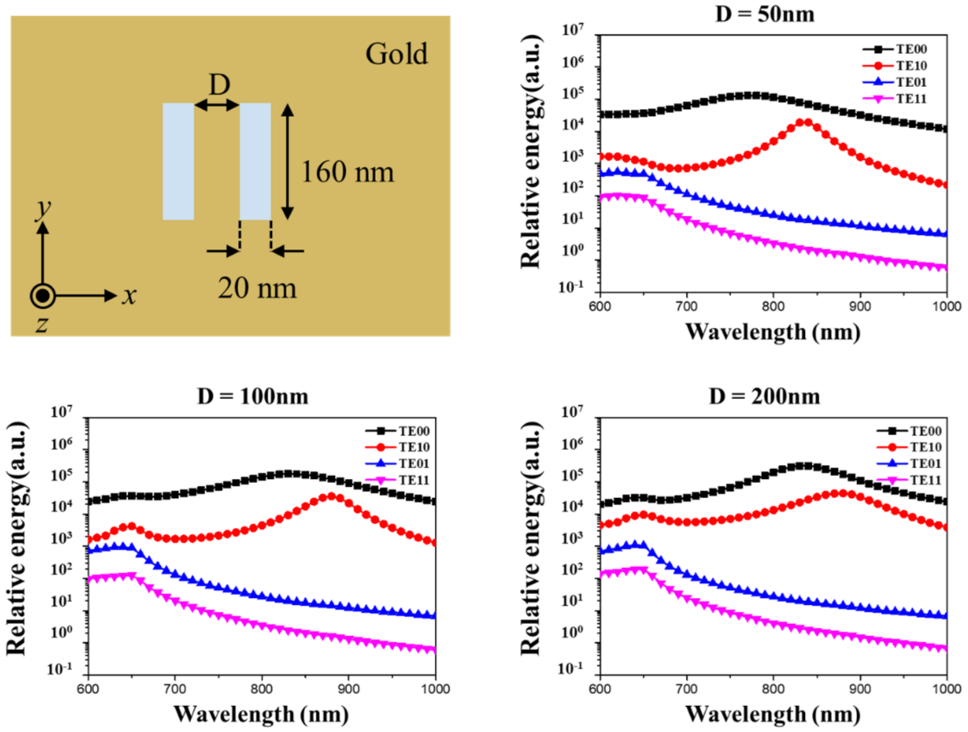

Figure S6. Unnormalized energy spectra of double-slot nanoantenna depending on slot spacing: Individual energy spectra for the slot spacing of double-slot nanoantennas: D= 50, 100, and 200 nm, respectively.

Supplementary Note 8: Mode decomposition using T-matrix with different number of input angles

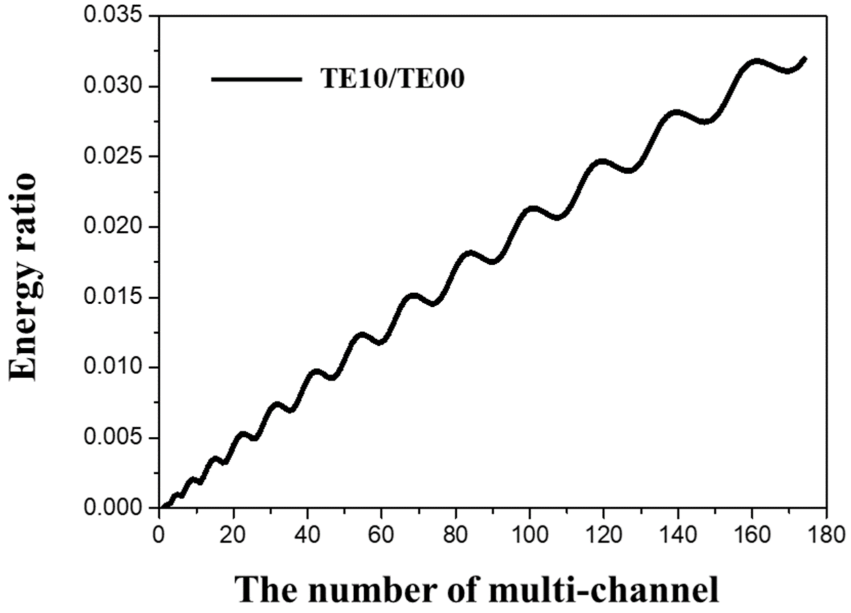

Figure S7. Mode decomposition using T-matrix with different number of input angles: Energy ratio of TE10 to TE00 mode resolved by T-matrix analysis as the number of incident beam angles increases.
